# Phytochemical screening and antibacterial potential of Peruvian propolis extract against Enterococcus faecalis: An in vitro study

**DOI:** 10.21142/2523-2754-1402-2026-283

**Published:** 2026-04-04

**Authors:** Andrea Yolanda Munsibay Foronda, Pamela Leonor Espinoza Salas, Amanda Lovera Arellano, Raúl Mauricio Olaechea Alejo, Cinthya Ruth Chipana Herquinio, Gabriel Nima

**Affiliations:** 1 Universidad Científica del Sur, Faculty of Health Sciences, Department of Dentistry. Lima, Peru. 100070348@cientifica.edu.pe, 100073084@cientifica.edu.pe, cchipanahe@cientifica.edu.pe, gabrieln_b@yahoo.com Universidad Científica del Sur Universidad Científica del Sur Faculty of Health Sciences Department of Dentistry Lima Peru 100070348@cientifica.edu.pe 100073084@cientifica.edu.pe cchipanahe@cientifica.edu.pe gabrieln_b@yahoo.com; 2 Universidad Científica del Sur, Faculty of Health Sciences, Department of Pharmacy and Bio-chemistry. Lima, Peru. alovera@cientifica.edu.pe Universidad Científica del Sur Universidad Científica del Sur Faculty of Health Sciences Department of Pharmacy and Bio-chemistry Lima Peru alovera@cientifica.edu.pe; 3 Laboratorio Húmedo Experimental, Universidad Científica del Sur. Lima, Peru. rolaecheaa@cientifica.edu.pe Universidad Científica del Sur Laboratorio Húmedo Experimental Universidad Científica del Sur Lima Peru rolaecheaa@cientifica.edu.pe

**Keywords:** anti-bacterial agents, *Enterococcus faecalis*, flavonoids, propolis, pulpectomy agent, agentes antibacterianos, *Enterococcus faecalis*, flavonoides, propóleos, agente para pulpectomía (DeCS)

## Abstract

**Objectives:**

This study aimed to obtain a propolis alcoholic extract (PAE), analyze its phytochemical screening, and test its antibacterial activity against *Enterococcus faecalis* to demonstrate its potential usage in pulp treatments for primary teeth.

**Materials and methods:**

PAE was obtained by maceration with absolute alcohol. The solubility assay and a phytochemical screening were performed. The phenolic compounds and flavonoids were quantified using spectrophotometry methods. The antibacterial activity of PAE was evaluated by determination of their minimal inhibitory concentration (MIC) and minimal bactericidal concentration (MBC) against *E. faecalis*. Two intracanal experimental propolis-based pastes were fabricated with propylene glycol and compared with the triple antibiotic paste (TAP) through the agar-well diffusion method against *E. faecalis*, and the results were analyzed by the Kruskal-Wallis test (a = 0.05).

**Results:**

The solubility test showed that ethanol was the best solvent for PAE. The phytochemical screening indicates the presence of tannins, alkaloids, flavonoids, anthocyanidins, aldehydes, glycosides, ketones, triterpenoids, steroids, and catechins in PAE. The phenolic and flavonoid content was 49.8 ± 0.13 GAE mg/ml and 0.824 ± 0.03 QE mg/ml, respectively. The results showed that PAE has antibacterial activity against *E. faecalis* with MIC and MBC values of 60 mg/ml and 130 mg/ml, respectively. The agar well diffusion assay showed that the propolis-based paste has antibacterial activity. However, TAP exhibited superior antibacterial activity against *E. faecalis*.

**Conclusion:**

PAE has phenolic and flavonoid content with antibacterial activity against *E. faecalis*. However, TAP exhibited higher antibacterial activity than PAE.

## RESUMEN

**Objetivos:**

El estudio tuvo como objetivo obtener un extracto alcohólico de propóleo (EAP), analizar su perfil fitoquímico y evaluar su actividad antibacteriana contra *Enterococcus faecalis*, considerando su posible uso en tratamientos pulpares de dientes primarios.

**Materiales y métodos:**

El EAP se obtuvo por maceración con alcohol absoluto. Se evaluó su solubilidad y perfil fitoquímico. Los compuestos fenólicos y flavonoides fueron cuantificados mediante métodos espectrofotométricos. La actividad antibacteriana frente a *E. faecalis* se determinó mediante la concentración mínima inhibitoria (CMI) y bactericida (CMB). Además, se elaboraron dos pastas experimentales con EAP y propilenglicol, y se las comparó con la pasta triple antibiótica (PTA) mediante difusión en agar. Los datos fueron analizados con la prueba de Kruskal-Wallis (α = 0,05).

**Resultados:**

El etanol fue el solvente más eficaz para el EAP. El perfil fitoquímico reveló la presencia de taninos, alcaloides, flavonoides, antocianinas, aldehídos, glucósidos, cetonas, triterpenoides, esteroides y catequinas. El contenido de compuestos fenólicos y flavonoides fue de 49,8 ± 0,13 mg EAG/ml y 0,824 ± 0,03 mg EQ/ml, respectivamente. El EAP mostró actividad antibacteriana contra *E. faecalis* con CMI de 60 mg/ml y CMB de 130 mg/ml. Las pastas con EAP mostraron efectividad antibacteriana; sin embargo, la PTA fue más eficaz.

**Conclusión:**

El EAP posee compuestos fenólicos y flavonoides con actividad antibacteriana frente a *E. faecalis*. No obstante, la PTA presentó mayor efectividad antibacteriana.

## INTRODUCTION

Pediatric pulp problems represent a commonly managed challenge in pediatric dentistry, presenting unique considerations distinct from those in adult patients. Pulp therapy often involves addressing issues such as root resorption, inadequate bone support, periodontal complications, presence of permanent tooth germs, and challenges related to patient cooperation ^1^. Given the crucial role of primary dentition in the development of occlusion, the focus of pulp therapy is oriented toward preserving primary teeth to ensure an optimal eruption sequence ^2^.

An approach for pulp therapy in primary teeth is the "Lesion Sterilization and Tissue Repair" (LSTR) technique. This technique involves minimal or non-instrumentation, followed by the application of a mixture of antibiotics in a vehicle, such as propylene glycol, in the root canal system ^2^^,^^3^. The LSTR technique allows the sterilization of the root canal system by significantly reducing microbial load, promoting tissue repair, and preventing the premature loss of primary teeth, thereby creating a favorable environment for natural tissue healing. In recent years, LSTR has gained attention for its potential to facilitate dentin remineralization and support the natural healing process in pediatric patients ^3^. The most common medicament used in LSTR is the TAP, which combines metronidazole, ciprofloxacin, and minocycline ^4^^,^^5^.

Triple antibiotic paste (TAP) provides broad-spectrum action for disinfecting primary teeth root canals. Metronidazole targets anaerobic bacteria, minocycline acts on gram-positive and gram-negative bacteria, and ciprofloxacin is effective against gram-negative bacteria ^6^. However, TAP presents drawbacks: (i) minocycline may cause tooth discoloration; (ii) risk of antibiotic resistance; and (iii) allergic reactions, including penicillin sensitivity ^6^. Additionally, minocycline can reduce dentin microhardness due to its chelating effect on the collagen matrix ^7^. In recent years, propolis has been increasingly valued in biomedicine for its antimicrobial activity.

Propolis is a sticky natural substance obtained by bees from flower resin, tree leaves, and plants mixed with their saliva ^8^. Propolis is composed of approximately 50% resin, 30% wax, 10% essential oils, 5% pollen, and other organic compounds ^9^. The antimicrobial activity of propolis is mainly attributed to its flavonoids and phenols content ^10^^,^^11^. Multiple investigations have confirmed the effectiveness of propolis against *Enterococcus faecalis*, a resistant bacterium often involved in endodontic infections ^12^^,^^13^. However, there is a lack of information regarding the use of propolis as a paste in the LSTR technique. 

Thus, the purpose of this study was to obtain an PAE, analyze its phytochemical profile, and test its antibacterial activity against *E. faecalis* to demonstrate its potential usage in pulp treatments for primary teeth. The hypotheses tested were that: (i) the PAE would indeed have phenolic compounds and flavonoids content, (ii) the PAE would have antimicrobial activity against *E. faecalis*, and (iii) there would be no differences among the PAE and TAP as an intracanal medicament regarding their antibacterial activity against *E. faecalis*.

## MATERIALS AND METHODS

### Propolis sample and preparation of alcoholic extract

The propolis was donated by the National Institute of Health-National Center for Social Research and Interculturality in Health, Lima, Peru. Propolis was collected from Tarapoto City, San Martín province, Peru, and stored at 4 °C until needed. No special permission was required for the Propolis collection. 

The collected propolis (200 gr) was manually ground and placed in a flask with 96% ethanol to obtain a 10% w/v solution. The solution was then subjected to an orbital shaker (ORBIT 1000, Labnet International, Edison, NJ, USA) at 25 °C and 200 rpm for 3 h, protected from light. The macerate was filtered through filter paper and stored for 7 days at room temperature in dark conditions; this extraction process was repeated twice.

The supernatant was carefully removed using a pipette, filtered through filter paper, and the ethanol was eliminated in a rotary evaporator (RV 10C, IKA-Werke GmbH, Staufen, Germany) (20 rpm, 100 bar, 40 °C, 4 h). The extract was collected, placed in a glass container, and stored (40 °C for 24 h) to ensure solvent removal. Finally, a high-viscosity resin was obtained and stored at 4 °C until needed.

### Bacterial strain and growth conditions

The experiments were conducted using *Enterococcus faecalis* (ATCC 29212), obtained from the “Laboratory of Microbiology and Molecular Genetics” at the XXXXXXXXX. The bacterial culture was stored at -80°C in Brain Heart Infusion (BHI) medium with 20% (v/v) glycerol. For reactivation, 50 µL of the glycerol stock was streaked on a Petri dish with BHI agar and incubated for 24 h at 37 ºC. The purity of the culture was confirmed using the Gram staining method.

### Solubility assay of propolis extract

A solubility test was conducted to identify the optimal solvent for the propolis extract. 1 mg of the alcoholic extract was diluted in 1 ml of nine different solutions: water, 70% alcohol, 96% alcohol, acetonitrile, dichloromethane, chloroform, methanol, 10% sodium hydroxide, and 10% hydrochloric acid. Subsequently, each tube was placed on a vortex shaker at 4200 rpm for 60 s.

### Phytochemical screening

The composition of propolis varies depending on factors such as the plant origin, geographic region, local flora, climate conditions, and the species of bees that collect it ^14^. Standard procedures were employed to conduct a phytochemical screening, assessing the presence or absence of biologically active compounds in the propolis extract, following the methodology described by Shaikh JR and Patil M ^15^. Table 1 shown the biologically active compounds studied.

**Table 1:** Qualitative test for phytochemical qualitative analysis (^15^)

Phytochemical Test	Test procedure	Observations	Presence
*Ninhydrin test*	1ml PAE + 2ml ninhydrin reagent + heating	Formation of blue color	Proteins
*Biuret test*	1ml PAE + 1ml biuret reagent	Formation of violet	Peptide linkage
*Molisch’s test*	2 ml PAE + 2 ml Molisch's reagent + few drops of sulfuric acid	Formation of purple or purplish-red ring	Carbohydrates
*Fehling’s test*	1 ml Fehling's reagent A + 1ml Fehling's reagent B + 1 ml PAE + heating	Fomation of reddish brown precipitate	Reducing sugars
*Kedde’s test*	1 ml Kedde's reagent A + 1ml Kedde's reagent B + 1 ml PAE	Formation of blue-violet	Cardiac Glycoside
Resin test	1 ml PAE + 5 ml destillied water + heating	Formation of precipitate at the bottom	Resins
*Sudan test*	1 ml PAE + 1 ml Sudan III dye + heating	Formation of red color	Lipids
*Liebermann-Buchart test*	1 ml PAE + Heat + Chloroform + few drops of sulfuric acid	Formation of green or green-blue color	Triterpenes and Steroids
*Gelatin test*	1 ml PAE + 1 ml gelatin solution + shaking	Formation of a white precipitate	Phenols
*Dragendorff’s test*	1 ml PAE + heating + 1 ml of 1% hydrochloric acid + six drops Dragendorff’s reagent	Formation of an orange-red precipitate	Alkaloids
*Mayer’s test*	1 ml PAE + heating + 1 ml of 1% hydrochloric acid+ Six drops of Mayer’s reagent	Formation of a white or cream precipitate	Alkaloids
*Shinoda’s test*	1 ml PAE + 1 ml hydrochloric acid + mixed + magnesium ribbon + amyl alcohol	Formation of pink coloration	Flavonoids
*Amylic test*	1 ml PAE + 0.5 ml hydrochloric acid + mixed + heating + 0.5 ml distilled water + 1 ml amyl alcohol + shaking	Formation of red or brown coloration	Anthocyanin
*Tollens’ test*	1 ml PAE + 1 ml tollens’ reagent + heating	formation of a silver-mirror precipitate	Aldehydes
Foam test	1 ml PAE + heating + 2 ml water + vigorously shaken (3 min)	Formation of foam	Saponins
*Bornträger reaction*	1 ml PAE + heating + chloroform + Bornträger’s reagent	*Formation of a red coloration*	Quinone
*Test for catechin*	Two drops of PAE + applied to filter paper water + few drops of sodium carbonate solution + exposed to UV light vigorously shaken (10 min)	Presence of a green stain under UV light	Catechin

### Total phenolic compounds content analysis

The phenolic content of the propolis alcoholic extract (PAE) was determined using the Folin-Ciocalteu method. A 2.6 ml reaction mixture was prepared with 0.2 ml of PAE (26.1 mg/ml), Folin-Ciocalteu reagent, sodium carbonate, and distilled water, then incubated in the dark at room temperature for 30 minutes. Absorbance was measured at 765 nm using a UV/Vis spectrometer (Genesys 10 UV, Thermo Scientific, Rochester, NY, USA). A gallic acid calibration curve (10, 25, 50, and 100 µl of 10 mg/ml solution) was used to quantify phenolics, with results expressed in mg of gallic acid equivalents per gram of PAE (mg GAE/g), based on the equation y = 0.0098x - 0.0035 (R² = 0.999). The analysis was performed in triplicate.

### Total flavonoid content analysis

The flavonoid content of the PAE was determined using quercetin as a standard. PAE (26.1 mg/ml) was diluted in 96% ethanol to 0.605 mg/ml, and absorbance was measured at 725 nm using a UV/Vis spectrometer. Quercetin standards (0.1, 0.2, and 0.3 mg/ml) in ethanol were used to generate a calibration curve. Results were expressed in mg of quercetin equivalent per gram of PAE (mg QE/g), based on the regression equation y = 0.0445x + 0.109 (R² = 0.9906). The analysis was conducted in triplicate.

### Minimum inhibitory concentration and minimum bactericidal concentration

For the experiments, 1 g of PAE was diluted in 2 ml of 70% ethanol, centrifuged (430 rpm, 3 min), filtered (0.22 µm), and stored at 4 °C in amber glass. Antimicrobial activity against *E. faecalis* was assessed by determining the Minimum Inhibitory Concentration (MIC) and Minimum Bactericidal Concentration (MBC). *E. faecalis* cultures were grown in BHI (14 h, 37 °C), adjusted to 0.5 McFarland, and used to inoculate 96-well plates containing PAE serially diluted (150-40 mg/ml). After 24 h incubation at 37 °C, the MIC was defined as the lowest concentration with no turbidity. To confirm, 50 µl of 0.1% resazurin (HiMedia Laboratories, Mumbai, India) was added, and plates were re-evaluated after 30 min in the dark. Color change indicated bacterial growth. For MBC, 10 µl from each well was plated on BHI agar and incubated (24 h, 37 °C). MIC and MBC tests were performed in triplicate.

### Agar well diffusion assay

To assess the antimicrobial activity of PAE, an agar well diffusion test was performed. Two experimental intracanal pastes containing PAE and propylene glycol were prepared based on the MIC and MBC results: one at the MBC concentration (130 mg/ml) and another 50% higher (200 mg/ml). These were compared with TAP (positive control) and propylene glycol alone (negative control). The groups were as follows:

• TAP: Ciprofloxacin (Clorfex 500 mg, InRetail Pharma, Madrid, Spain), minocycline (Acnebiot 100 mg, InRetail Pharma, Madrid, Spain), and metronidazole (Flagimed 500 mg, InRetail Pharma, Madrid, Spain), mixed 1:1:1 with propylene glycol (Farmacia Universal, Lima, Peru) as vehicle, following Hoshino et al.
• PE1: PAE (130 mg/ml) with propylene glycol.
• PE2: PAE (200 mg/ml) with propylene glycol.
• CON: Propylene glycol only.

PAE and propylene glycol were measured with micropipettes (ISOLAB Laborgeräte GmbH, Eschau, Germany), and antibiotics were weighed using an analytical balance (Pioneer Analytical PX224, Ohaus, Parsippany, NJ, USA). All pastes were freshly prepared.

A 50 µl aliquot of *E. faecalis* culture was spread on BHI agar plates (n=5) using a sterilized Drigalski spatula. After 5 minutes, four wells (5 mm diameter, 4 mm depth) were made in the agar and filled with 50 µl of each paste. Plates were incubated at 37 °C for 24 h.

Antibacterial activity was assessed by measuring the inhibition zones (in mm) from the well edge to the nearest bacterial growth. Plates were photographed individually, and inhibition zones were measured using ImageJ (US NIH, Bethesda, Maryland, USA). The test was conducted in triplicate.

### Statistical analysis

The data from the agar well diffusion assay was tested for normality and homogeneity of variances using the Shapiro-Wilk and Bartlett tests (α = 0.05). The results indicated that the data did not follow a normal distribution (p<0.05). Subsequently, a Kruskal-Wallis test (α = 0.05) was employed to analyze statistical differences between groups. The data analysis was conducted using STATA 17.0 (Stata Corporation, Texas, USA).

## RESULTS

The solubility of propolis was tested in water and various organic solvents, with the results presented in Table 2. The results indicated that propolis exhibited the highest solubility in 96º ethanol, dichloromethane, and methanol. In contrast, no solubility was observed in sodium hydroxide (10%) and hydrochloric acid (10%).

**Table 2:** Solubility test of propolis extract in various solvents

Solvents	Solubility index*
Distilled water	+
Ethanol 70º	+
Ethanol 96º	+++
Acetonitrile	+
Dichlorometane	+++
Chloroform	++
Methanol	+++
Sodium hydroxide 10%	-
Hydrocloridic acid 10%	-

* (-) No dissolution was observed; (+) A small amount of propolis was dissolved. Particle agglomeration was seen at the bottom of the tube; (++) Some particles were dissolved in the solvent, but the precipitate indicates partial or total fragmentation; (+++) Most particles were dissolved in the solvent.

Phytochemical screening of the propolis extract involved testing for 16 chemical compounds. The reactions with various reagents revealed the presence or absence of the investigated compounds, as presented in Table 3. Phytochemical screening identified lipids, tannins, and phenolic compounds, including flavonoids, among other compounds. Conversely, amino acids, saponins, and quinones, among others, were not detected.

**Table 3:** Phytochemical screening of the propolis extract

+Chemical class	Method	Result*
Amino acids	Ninhidrina	-
Proteins	Biuret	-
Carbohydrates	Molish	-
Reducing sugars	Feeling	-
Cardiotonic glycosides	Kedde	+
Resins	Distilled water	+
Lipids	Sudan	+
Tannins	Gelatin test	+
Alkaloids	Dragendorff	+
Alkaloids	Mayer	+
Flavonoids	Shinoda	+
Anthocyanidins	Amyl acid	+
Aldehydes and ketones	Tollens	+
Saponinas	Foam test	-
Triterpenoids and steroids	Lieberman-Buchard	+
Quinones	Bonträger	-
Catechins	Sodium carbonate	+

* +: presence, -: absence.

**Figure 1:**
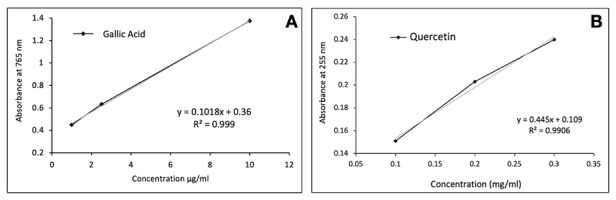
Calibration curves. (A) Calibration curve of gallic acid for determining the total phenolic content, and (B) Calibration curve of quercetin for determining the total flavonoid content.

Phenol compounds content was determined to be 49.8 ± 0.13 GAE mg/ml of PAE and 0.824 ± 0.03 QE mg/ml of PAE for flavonoids (Figure 1).

**Figure 2:**
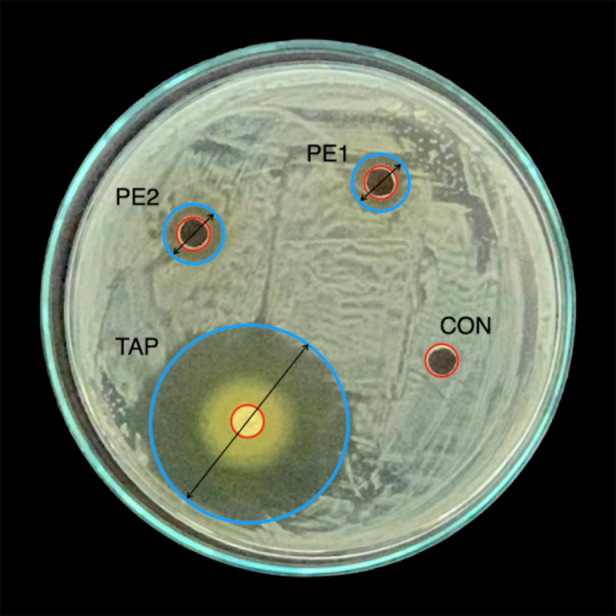
The Petri dish displays the antimicrobial activity of the intracanal pastes against E. faecalis. CON: Propylene Glycol, TAP: Triple antibiotic paste, PE1: PAE paste at 130 mg/ml, and PE2: PAE paste at 200 mg/ml.

The antimicrobial properties of the propolis extract against *E. faecalis* were investigated, determining the MIC at 60 mg/ml and the MBC at 130 mg/ml. The agar well diffusion assay exhibited a potent bacterial effect for the TAP, with statistically significant differences compared to 200 mg/ml and 130 mg/ml propolis-based pastes (Figure 2). 

Table 4 shows that there are no statistically significant differences between the propolis-based paste formulations.

**Table 4:** Diameter of inhibition zone in millimeters of the experimental intracanal pastes against *E. faecalis* (n = 15)

Group	Median [IQR]
CON	0 [0] C
PE1	8.1 [3.1] B
PE2	8.5 [2.9] B
TAP	36.0 [9.5] A

*Medians followed by different letters indicate statistical differences. Kruskal-Wallis test (a = 0.05). ** CON: Propylene Glycol, TAP: Triple antibiotic paste, PE1: PAE at 130 mg/ml, and PE2: PAE at 200 mg/ml.

## DISCUSSION

Propolis is a resinous substance that possesses various properties, including anti-tuberculosis, anti-inflammatory, antiviral, and antioxidant ^16^^,^^17^, with several studies demonstrating its antibacterial activity ^8^^,^^18^^-^^20^. In this study, an ethanolic propolis extract (PAE) was prepared, followed by phytochemical analysis, determination of minimum inhibitory concentration (MIC), minimum bactericidal concentration (MBC), and an agar well diffusion assay. The extract showed a high concentration of phenolic compounds and flavonoids, confirming its antibacterial efficacy against *E. faecalis*. 

The solvent used to prepare the PAE could influence its biomedical properties ^17^. Ethanol was chosen over other solvents due to its proven effectiveness in dissolving active compounds like phenols and flavonoids, as supported by previous studies ^19^^, 21)^. A solubility test demonstrated that 96% ethanol, dichloromethane, and methanol effectively dissolved the extract. Ethanol was preferred for its ease of evaporation, cost-effectiveness, and ability to preserve and potentially enhance the antibacterial activity of the extract ^21^. However, its use raises safety concerns in vulnerable groups such as pregnant women and children, which has prompted interest in identifying safer alternative solvents ^19^^,^^22^.

Pharmaceutical use of propolis is increasing, with various products available, though its clinical effectiveness remains uncertain. The composition and properties of propolis depend on solvents and extraction methods like ethanol, water, and ultrasound-assisted techniques. Standardized extraction methods are needed, with ultrasound-assisted extraction often yielding optimal results. Guidelines for quality control and further studies on propolis constituents are necessary to understand its mechanisms. Standardization will improve production consistency and clinical applications ^23^.

The properties of propolis are intricately linked to the bee race, geographical area of resin collection, botanical source ^24^, and even the season of collection ^8^^,^^25^^, 26)^, leading to variability in its bioactive compounds. A study compared the antioxidant activity of propolis samples originating from distinct geographical regions and plant sources; demonstrating that the propolis's antioxidant activity is correlated with both its origin and its phenolic compounds ^24^. Bueno-Silva et al. collected red propolis over the course of a year, comparing its antibacterial activity across different seasons. Their study demonstrated that the season in which propolis and its botanical source are harvested leads to variations in its antibacterial properties ^26^. Another study investigated the antibacterial, antibiofilm, cytotoxic activities, and chemical compositions of 13 Peruvian propolis samples against oral biofilms, revealing variations in antibacterial activity and demonstrating differences in bioactive compounds ^14^. The propolis used in this study was collected from Tarapoto City, San Martín province, Peru, where the flora is responsible for the concentration of bioactive compounds found in the extract used in this research. 

In this study, the phytochemical analysis of PAE revealed the presence of alkaloids, resins, aldehydes, ketones, triterpenes, steroids, phenols, and flavonoids. The combination of these and other compounds is responsible for the bioactive properties of propolis and is directly related to the factors mentioned previously ^16^. Additionally, the lack of standardized methods and analyses to evaluate its composition complicates the understanding of its bioactive properties and the compounds related to each of them even more ^27^.

The results revealed that the PAE contains phenolic compounds and flavonoids in its composition. Thus, the initial hypothesis, stating that the alcoholic extract of propolis would indeed have phenolic compounds and flavonoids content, was confirmed. Phenolic compounds and flavonoids exhibit diverse therapeutic properties and demonstrate strong antibacterial activity and could be considered an indicator to assess the propolis’ quality ^17^^,^^24^^,^^28^. Phenolic compounds also shown antioxidant properties, effectively combating oxidative stress and inflammation ^18^. Additionally, flavonoids, as a subclass of phenolic compounds, exhibit remarkable antibacterial activity by disrupting bacterial cell membranes and inhibiting microbial growth ^12^. 

Various mechanisms of action have been proposed for the antibacterial activity of propolis, including the inhibition of nucleic acid synthesis, alteration of cytoplasmic membrane function, inhibition of energy metabolism, reduction of biofilm development, inhibition of cell membrane proteins, and increased membrane permeability ^29^. The efficacy of phenolic and flavonoid compounds is influenced by their composition, structure, and the specific bacterial strains targeted ^8^. Therefore, a single mechanism for the antibacterial action of propolis remains to be definitively determined ^8^.

The collective action of compounds found in propolis provides a natural alternative to conventional antibiotics ^19^. In this study, phenolic compounds and flavonoids found in PAE (Fig. 1) exhibit variations when compared with other studies, as expected. As mentioned earlier, propolis can display differences in phytochemical profiles based on factors such as the season and location of collection, as well as the extraction methods used, etc. ^18^. Hernández et al. identified significant variability in phenolic compounds (ranging from 68 to 500 QE mg/g) and flavonoids (caffeic acid equivalents from 13 to 379 mg/g) in the alcoholic extract of propolis ^28^. Similarly, another study analyzed propolis from various regions of Poland, revealing high variability in the content of phenols (116.16 - 219.41 mg GAE mg/g) and flavonoids (29.63-106.07 QE mg/g) ^30^. Additional studies have demonstrated considerable variability in propolis compounds ^14^^,^^16^^,^^30^. According to Paula et al., the method used to analyze propolis also influences the variability of propolis compounds found in the literature ^31^. In this context, it is essential to acknowledge that the bioactive properties in propolis arise from the combination of various bioactive compounds, including phenolic compounds ^31^. Therefore, there isn't a universally defined minimum acceptable level for phenolic compounds and flavonoids in propolis.

The PAE demonstrated antibacterial activity against *E. faecalis*, confirming the acceptance of the second hypothesis, stating that the alcoholic extract of propolis would exhibit antimicrobial activity against *E. faecalis*. In previous studies, MIC values ranged from 2 µg/ml to 1000 mg/ml ^20^, and the value obtained in this research falls within this intermediate range. The variations in MIC values can be attributed to factors such as the methodology employed, experimental design, and the origin of the propolis. These elements contribute significantly to the divergent MIC values observed across multiple studies.

In this study, two experimental pastes based on propolis and using propylene glycol as a vehicle, were prepared. The selection of an appropriate vehicle is crucial, as it can influence the properties of the active principle when mixed with the vehicle, either enhancing or limiting its effectiveness. Propylene glycol is widely used as a vehicle in endodontics due to its low toxicity and high biocompatibility, which result in minimal adverse effects on periapical tissues. Additionally, its low viscosity and high penetrability contribute to its favorable characteristics. Functioning as a hydrophilic solvent, propylene glycol aids in the efficient dissolution of various medicaments, guaranteeing their uniform distribution within the root canal system. While some studies have attributed antimicrobial properties to propylene glycol, it is remarkable that in our study, no antimicrobial activity was observed ^32^.

The results of the well diffusion agar test demonstrated a reduced antibacterial activity for the two experimental pastes in comparison to TAP. These outcomes reject the third null hypothesis, stating that there would be no differences among the alcoholic extract of propolis and the TAP as intracanal medicaments in primary teeth regarding their antibacterial activity against *E. faecalis*. At this respect, it is essential to note, that these results may be influenced by the test employed, as the well diffusion agar test exhibits limitations when applied to natural extracts. 

The most significant limitations of the agar diffusion test are associated with the complex composition of the natural extracts. Among these limitations, the following reasons can be highlighted: (i) limited and irregular diffusion rates of bioactive compounds. Due to the complex composition of the natural extract, some compounds may experience limited penetration or exhibit varying diffusion rates, resulting in irregular zones of inhibition and inaccurate results; (ii) interference with the agar composition, which affects the diffusion process and leads to erroneous results; and (iii) the lack of standardization of the agar medium, making comparison with other studies challenging ^27^. These limitations could have influenced the outcomes obtained in this study, potentially reducing the antibacterial activity of propolis.

The results of this study confirm that propolis exhibits antibacterial activity against *E. faecalis* and could serve as an alternative intracanal paste for pulpectomy treatments. Additionally, propolis holds potential for various applications, such as disinfecting cavity preparations and root surfaces, providing the advantage of using a material with unspecific antibacterial mechanisms ^8^. Regarding the experimental design, despite the use of bacteria in a different form than they are typically found in pulp pathologies, which may limit clinical relevance, this study can be viewed as a proof-of-concept effort to assess the antibacterial activity of propolis and identify active components in the alcoholic extract. Future studies utilizing propolis extract could take a more clinically oriented approach, avoiding the use of an agar model and opting for a model that better replicates oral conditions. Further studies on propolis should explore additional properties to ensure its clinical applicability, including biocompatibility, interactions with dentin structure, and the longevity of its effects.

Propolis displayed a significant concentration of phenolic compounds and total flavonoids, emphasizing its potential as a rich source of bioactive compounds. Moreover, the PAE exhibited antimicrobial activity against *E. faecalis*. However, the antibacterial efficacy demonstrated by the PAE paste was found to be reduced compared to the TAP, likely influenced by the experimental design employed.

The results suggest that PAE could serve as a viable alternative to conventional intracanal pastes. Nevertheless, further studies are necessary to validate and optimize its use in pulpectomy treatments.

## CONCLUSION

Propolis showed a high concentration of phenolic compounds and total flavonoids, underscoring its potential as a rich source of bioactive molecules. Additionally, the PAE demonstrated antimicrobial activity against E. faecalis. Nevertheless, its lower antibacterial efficacy compared to TAP may be attributed to methodological limitations, highlighting the need for further investigation. More clinically oriented studies are required to assess its biocompatibility and long-term stability, thereby validating and optimizing propolis as a potential intracanal medicament for pulpectomy procedures.
